# Full-Reference Image Quality Assessment with Linear Combination of Genetically Selected Quality Measures

**DOI:** 10.1371/journal.pone.0158333

**Published:** 2016-06-24

**Authors:** Mariusz Oszust

**Affiliations:** Department of Computer and Control Engineering, Rzeszow University of Technology, Rzeszow, Poland; Nankai University, CHINA

## Abstract

Information carried by an image can be distorted due to different image processing steps introduced by different electronic means of storage and communication. Therefore, development of algorithms which can automatically assess a quality of the image in a way that is consistent with human evaluation is important. In this paper, an approach to image quality assessment (IQA) is proposed in which the quality of a given image is evaluated jointly by several IQA approaches. At first, in order to obtain such joint models, an optimisation problem of IQA measures aggregation is defined, where a weighted sum of their outputs, i.e., objective scores, is used as the aggregation operator. Then, the weight of each measure is considered as a decision variable in a problem of minimisation of root mean square error between obtained objective scores and subjective scores. Subjective scores reflect ground-truth and involve evaluation of images by human observers. The optimisation problem is solved using a genetic algorithm, which also selects suitable measures used in aggregation. Obtained multimeasures are evaluated on four largest widely used image benchmarks and compared against state-of-the-art full-reference IQA approaches. Results of comparison reveal that the proposed approach outperforms other competing measures.

## Introduction

Visual information is often a subject of many processing steps, e.g., acquisition, enhancement, compression, or transmission. After processing, some information carried by the content of the image can be distorted. Therefore, its quality should be evaluated from a human perception point of view. There are three categories of image quality assessment (IQA) measures (metrics or models), depending on availability of a pristine, i.e., distortion-free, image: (1) full-reference, (2) no-reference, and (3) reduced-reference models. In this paper, the full-reference approach is considered, in which for each distorted image in a benchmark dataset its reference image is provided.

Application of peak signal-to-noise ratio (PSNR) is one of the simplest approaches to IQA. However, an output of PSNR is not well correlated with human evaluation; therefore this technique often serves as a bottom model for comparison. In [1], Damera-Venkata et al. presented noise quality measure (NQM) in which a distorted image is modelled using a linear frequency distortion and an additive noise injection. Wang et al. [2] introduced universal image quality index (UQI). UQI evaluates quality of an image using loss of correlation, luminance distortion, and contrast distortion. Further extension of UQI, structural similarity (SSIM), was proposed by Wang et al. [3]. A multi-scale SSIM, MSSIM, was presented in [4]. Wang and Li in [5] proposed information content weighted SSIM (IW-SSIM) approach as an extension of MSSIM. In that work, local information was measured using statistical models of natural scenes. Statistical properties of natural environment are also utilised in visual information fidelity (VIF) [6] measure and information fidelity criterion (IFC) [7]. In [8], Riesz-transform based feature similarity (RFSIM) was proposed. The measure is computed by comparing Riesz-transform features at key locations between the distorted image and its reference image. Authors of feature similarity index (FSIM) [9] developed an approach which uses phase congruency and image gradient magnitude as low-level local features. FSIMc is a version of FSIM developed for processing colour images. In [10], spectral residual based similarity (SR-SIM) using visual saliency map was proposed. A visual saliency to calculate a local quality map of the distorted image is used in visual saliency-induced index (VSI) [11]. The gradient similarity (GSM) measure [12] estimates image quality taking into consideration structure and contrast changes, as well as luminance distortions. In [13], image structural degradation was considered and determined using local binary patterns. In SURF-SIM [14], multiscale differences between features detected and described by Speed Up Robust Features (SURF) approach are combined with a pooling strategy. An IQA measure that evaluates images taking into account inter-patch and intra-patch similarities was described in [15]. In that work, authors used modified normalised correlation coefficient and image curvature.

Development of full-reference IQA measures can also involve different fusion strategies. For example, Liu and Yang [16] combined SNR, SSIM, VIF, and VSNR using canonical correlation analysis. A most apparent distortion algorithm (MAD) [17] adopts two strategies for IQA. In that approach, a local luminance and a contrast masking evaluate high-quality images. Changes in the local statistics of spatial-frequency components are used for images with a low quality. Three IQA metrics, MS-SIM, VIF and R-SVD, were non-linearly combined by Okarma in [18, 19]. A non-linear fusion of IQA measures was also investigated in [20]. In [21], up to seven IQA models were combined using a regularised regression. Peng and Li in [22] presented an approach based on conditional Bayesian mixture of experts model. In that paper, a support vector machines classifier was used for prediction of the type of distortion, and then SSIM, VSNR, and VIF with k-nearest-neighbour regression were fused. Authors in their other paper, [23], presented and adaptive combination of IQA measures with an edge-quality based on preservation of edge direction. In [24], a combination of local and global distortion measures was considered using saliency maps, gradient and contrast information.

Recently, many complex fusion approaches have been introduced, and therefore, the main contribution of this paper is to show that a solution based on linear combination, which, together with a genetic algorithm, is able to find well-performing fusion of IQA measures. Apart from comparison of different approaches performed in accordance to a widely accepted protocol, the paper provides some insights on a selection of IQA techniques which are likely to be fused. In this paper, a decision fusion of 16 full-reference IQA measures is defined as an optimisation problem of finding weights in a weighted sum of their outputs. A genetic algorithm finds the solution that minimises root mean square error (RMSE) of prediction performance. The number of used measures and parameters of the regression model for fitting objective scores to subjective scores prior to RMSE calculation are found by the algorithm. Finally, the proposed approach is evaluated on four largest IQA image benchmarks and compared with the state-of-the-art approaches.

The rest of this paper is organised as follows. In the section Methods, a formulation of the optimisation problem and the development of the proposed approach are presented. Experimental results with related discussions are covered in the section Results and Discussion. Finally, the last section concludes the paper.

## Methods

Since digital processing can alter an appearance of the image and that may lead to different opinions on its quality, many IQA algorithms have been proposed for automatic assessment [25]. In order to compare IQA approaches, specific image databases have been proposed. They contain reference images, their corresponding distorted images, and ground-truth information obtained from human observers. Information on the perceived quality is reported as mean opinion scores (MOS values) or differential mean opinion scores (DMOS values).

The desired IQA metric should produce objective scores which are consistent with human ratings (subjective scores). In this work, it is assumed that joint metric can provide better results, in terms of prediction quality, than a single metric that contributes to the multimeasure.

Let *Q* be an output of an aggregated decision of *n* IQA measures, where *n* ∈ *N*. It can be expressed as:

$$\begin{array}{c} Q=A(Q_{1},Q_{2},\cdots ,Q_{n}), \end{array}$$ (1)

where *A* is an aggregation operator. The operator often has a form of a weighted sum [26–28], therefore *Q* can be expressed as follows:

$$\begin{array}{c} Q=\sum_{i=1}^{n}x_{i}Q_{i}, \end{array}$$ (2)

where **x** = [*x*_1_, *x*_2_, …, *x*_*n*_] denotes a vector of weights, $x\in R^{n}$. The vector **x** contains decision variables in an optimisation problem of finding an effective fusion of IQA measures. Since many fusions can be proposed, a given **x** should be evaluated. For this purpose one of typically used IQA measures quality evaluation indices can be used. In order to measure consistency of the output of the examined IQA model with human assessment, the following indices of prediction accuracy, monotonicity, and consistency are often considered [29, 30]: Spearman Rank order Correlation Coefficient (SRCC), Kendall Rank order Correlation Coefficient (KRCC), Pearson linear Correlation Coefficient (PCC), and Root Mean Square Error (RMSE). Evaluation indices are calculated after a nonlinear mapping between a vector of objective scores, ***Q***, and MOS or differential MOS (DMOS), ***S***, using the following mapping function for the nonlinear regression [30]:

$$\begin{array}{c} Q_{p}=F\left(Q,\beta \right)=\beta_{1}(\frac{1}{2}-\frac{1}{\text{exp}(\beta_{2}\left(Q-\beta_{3}\right))})+\beta_{4}Q+\beta_{5}, \end{array}$$ (3)

where ***β*** = [*β*_1_, *β*_2_, …, *β*_5_] are parameters of the regression model [29], and ***Q***_***p***_ is a mapped equivalent of ***Q***. SRCC is calculated as follows:

$$\begin{array}{c} \text{SRCC}\left(Q,S\right)=1-\frac{6\sum_{i=1}^{m}d_{i}^{2}}{m(m^{2}-1)}, \end{array}$$ (4)

where *d*_*i*_ is the difference between *i*^*th*^ image in ***Q*** and ***S***, and *m* is the total number of images. KRCC, in turn, uses the number of concordant pairs in the dataset, *m*_*c*_, and the number of discordant pairs in the dataset, *m*_*d*_. It is illustrated by Eq (5).

$$\begin{array}{c} \text{KRCC}\left(Q,S\right)=\frac{m_{c}-m_{d}}{0.5m(m-1)}. \end{array}$$ (5)

PCC is defined as:

$$\begin{array}{c} \text{PCC}\left(Q_{p},S\right)=\frac{{\bar{Q_{p}}}^{T}\bar{S}}{\sqrt{{\bar{Q_{p}}}^{T}\bar{Q_{p}}{\bar{S}}^{T}\bar{S}}}, \end{array}$$ (6)

where, $\bar{Q_{p}}$ and $\bar{S}$ denote mean-removed vectors. RMSE is given by Eq (7).

$$\begin{array}{c} \text{RMSE}\left(Q_{p},S\right)=\sqrt{\frac{{\left(Q_{p}-S\right)}^{T}\left(Q_{p}-S\right)}{m}}. \end{array}$$ (7)

Higher SRCC, KRCC, and PCC values are considered better, in contrary to the values of RMSE.

One of these performance indices could be used as an objective function in a considered optimisation problem. Preliminary experiments revealed that maximisation of SRCC or KRCC may lead to fusion providing unacceptably high RMSE values. On the other hand, RMSE requires determination of ***β***. Finally, RMSE was used as the objective function in the considered problem (Eq (8)), and ***β*** components were considered as decision variables in addition to the weights of fused IQA measures.

$$\begin{array}{cccc} & \underset{x}{\text{minimise}} & & \text{RMSE}(F(Q,\beta ),S)\\ & \text{subject}\;\text{to} & & x_{i}\in R,\;n\in N,\;\beta \geq 0 \end{array}$$ (8)

Linear combination may produce negative weights which can be unintuitive in terms of contribution of IQA measures that take part in the aggregation. Therefore, different combination types were considered starting from convex combination, in which weights are positive and their sum is equal one, affine combination with preserved sum condition, or conical combination with positive weights. Preliminary results confirmed that the proposed approach provides best performance without constraining the weights.

In this paper, an optimisation-based fusion was performed using *N* = 16 IQA measures with publicly available source code. The following techniques were used: VSI [11], FSIM [9], FSIMc [9], GSM [12], IFC [7], IW-SSIM [5], MAD [17], MSSIM [4], NQM [1], PSNR [29], RFSIM [8], SR-SIM [10], SSIM [3], VIF [6], IFS [31], and SFF [32]. In the proposed approach, the vector of decision variables, **x**, is obtained in a data-driven fashion. Since there are four largest widely used IQA image benchmarks, in this paper four IQA fusion measures are introduced. For this purpose, 20% of the reference images from the given dataset along with their distorted counterparts were used for training. In the literature, sometimes more images were utilised in order to tune parameters of developed methods, e.g., 30% [9, 11], 80% [13], or parameters were generated for each image dataset separately [13, 21–23]. Some approaches used images from all datasets for this purpose [15]. In order to show dataset-independent results, each fusion measure developed in this paper was evaluated on all datasets.

Finally, the vector $x_{d}^{best}$, where *d* denotes a dataset, was obtained in the following steps: (1) Selection of the 20% reference images from a given dataset and their distorted equivalents; (2) Evaluation of images using *N* = 16 full-reference IQA measures; (3) Selection of *n* ∈ *N* IQA measures, finding weights of linear combination of their opinion scores and ***β***. Objective scores of used measures, if needed, were scaled to be in a 0-1 range.

The optimisation problem was solved using a genetic algorithm (GA) [28, 33], since the number of possible solutions grows exponentially with the number of used IQA metrics. The GA uses a population of individuals, where each individual represents a single solution. Then, from generation to generation, after applying selection, crossover and mutation operators, better solutions are emerging. The GA was run for 200 generations, with a population of 100 individuals, elite count equal to 0.05 of the population size, and 0.8 crossover fraction. Scattered crossover, Gaussian mutation and stochastic uniform selection rules were used [33]. All presented calculations were performed using Matlab software (version 7.14) with GA Toolbox [34]. After 100 runs, the best solution, $x_{d}^{best}$, was selected. The individual in the proposed solution is represented by real-valued vector, where dimensions refer to weights of IQA measures, **x**, and ***β*** values. Parameters of the GA were determined experimentally observing convergence of the objective function over the generations.

**Fig 1:**
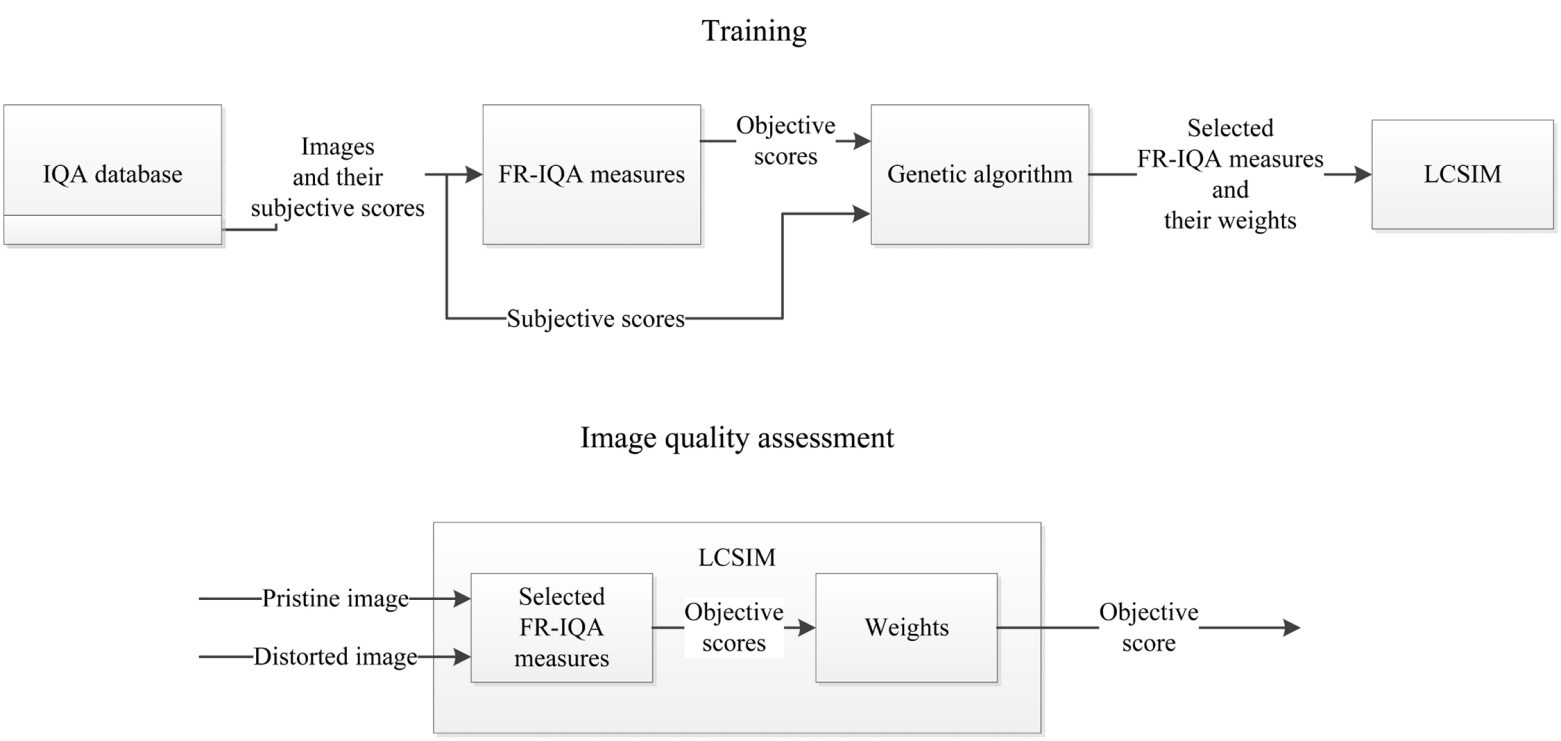
Flowchart of the proposed approach. In an offline training process, the proposed approach is obtained using some of images from a benchmark dataset. Images are assessed by full-reference IQA measures. Then, a genetic algorithm selects IQA measures and assigns weights to them. Obtained weights for linear combination of selected measures are used in image quality assessment tasks.

Fig 1 presents a flowchart of the approach with a process in which the introduced fusion measure is obtained and its usage for image quality assessment.

In experiments, the following four image benchmarks were used: TID2013 [35], TID2008 [36], CSIQ [17], and LIVE [3]. The number of reference images, distortions, and subjects for each dataset are shown in Table 1. Each database contains reference images, their corresponding distorted images and subjective scores.

**Table 1:** IQA benchmark image datasets.

Dataset	No. of reference images	No. of distorted images	No. of distortions	No. of observers
**TID2013** [35]	25	3000	24	971
**TID2008** [36]	25	1700	17	838
**CSIQ** [17]	30	866	6	35
**LIVE** [3]	29	779	5	161

Finally, four IQA measures, namely Linearly Combined Similarity Measures (LCSIMs), were obtained:

$$\begin{array}{ccc} \text{LCSIM1}=x_{TID2013}^{best} & = & 24.9166\text{VSI}-1.1853\text{FSIM}+7.2735\text{FSIMc}\\ & & -\;1.0367\text{IW-SSIM}-1.3816\text{MAD}-2.5171\text{MSSIM}\\ & & -\;0.8420\text{PSNR}+6.1916\text{SR-SIM}+5.6767\text{IFS}, \end{array}$$ (9)

$$\begin{array}{ccc} \text{LCSIM2}=x_{TID2008}^{best} & = & -14.0578\text{VSI}-2.4925\text{IW-SSIM}+4.5530\text{MAD}\\ & & +\;3.4421\text{MSSIM}-3.7502\text{PSNR}-0.7722\text{RFSIM}\\ & & -\;10.9245\text{SR-SIM}+5.6138\text{SSIM}-6.1125\text{VIF}-7.6139\text{IFS}, \end{array}$$ (10)

$$\begin{array}{ccc} \text{LCSIM3}=x_{CSIQ}^{best} & = & 2.1056\text{VSI}-5.5300\text{FSIM}+0.1928\text{FSIMc}\\ & & +\;6.4580\text{GSM}-9.2595\text{MAD}-10.7249\text{MSSIM}\\ & & +\;2.8804\text{RFSIM}+9.8189\text{SR-SIM}+4.4817\text{VIF}\\ & & +9.4591\text{IFS}+13.7206\text{SFF}, \end{array}$$ (11)

$$\begin{array}{ccc} \text{LCSIM4}=x_{LIVE}^{best} & = & -2.5748\text{FSIM}+12.3199\text{GSM}+1.1860\text{IFC}\\ & & +\;1.3884\text{IW-SSIM}+7.7745\text{MAD}+1.4430\text{NQM}\\ & & +\;0.6367\text{RFSIM}-5.3659\text{VIF}-2.8739\text{IFS}. \end{array}$$ (12)

Their corresponding ***β*** components are as follows:

$$\begin{array}{ccc} \beta_{\text{LCSIM1}} & = & [13.0262,0.1286,19.4659,1.4283,12.8159],\\ \beta_{\text{LCSIM2}} & = & [9.8714,6.2907,4.2059,8.1359,12.6196],\\ \beta_{\text{LCSIM3}} & = & [7.9955,7.1928,15.2802,5.0426,3.4213],\\ \beta_{\text{LCSIM4}} & = & [8.3862,8.0861,9.0601,11.5365,13.3231]. \end{array}$$

## Results and Discussion

This section presents experimental evaluation of the proposed approach in comparison with state-of-the-art techniques, as well as discussion on influence of the aggregated IQA measures and ***β*** on resulting fusion models.

### Comparative evaluation

For evaluation, four largest image benchmarks (TID2013, TID2008, CSIQ, and LIVE) and four performance indices (SRCC, PCC, KROCC, RMSE) were used.

Table 2 presents evaluation results for the best ten models and LCSIMs. The top two models for each criterion are shown in boldface. The table also contains direct and weighted averages of obtained values. For the weighted average, the number of images in the database is used as its weight. Overall results for RMSE do not take into account LIVE dataset due to range difference.

**Table 2:** Performance comparison of resulted fusion measures with IQA models that were used in optimisation.

	VSI	FSIM	FSIMc	GSM	MAD	MSSIM	SR-SIM	VIF	IFS	SFF	LCSIM1	LCSIM2	LCSIM3	LCSIM4
**SRCC**	**0.8965**	0.8015	0.8510	0.7946	0.7807	0.7859	0.7999	0.6769	0.8697	0.8513	**0.9044**	0.8139	0.8307	0.8086
**KRCC**	**0.7183**	0.6289	0.6665	0.6255	0.6035	0.6047	0.6314	0.5147	0.6785	0.6581	**0.7326**	0.6387	0.6534	0.6292
**PCC**	**0.9000**	0.8589	0.8769	0.8464	0.8267	0.8329	0.8590	0.7735	0.8791	0.8706	**0.9140**	0.7993	0.8659	0.8651
**RMSE**	**0.5404**	0.6349	0.5959	0.6603	0.6975	0.6861	0.6347	0.7856	0.5909	0.6099	**0.5030**	0.7449	0.6201	0.6218
TID2008														
**SRCC**	0.8979	0.8805	0.8840	0.8504	0.8340	0.8542	0.8913	0.7491	0.8903	0.8767	0.9057	**0.9178**	**0.9107**	0.8892
**KRCC**	0.7123	0.6946	0.6991	0.6596	0.6445	0.6568	0.7149	0.5860	0.7009	0.6882	0.7271	**0.7495**	**0.7391**	0.7053
**PCC**	0.8762	0.8738	0.8762	0.8422	0.8306	0.8451	0.8866	0.8084	0.8810	0.8817	0.8956	**0.9202**	**0.9113**	0.8965
**RMSE**	0.6466	0.6525	0.6468	0.7235	0.7473	0.7173	0.6206	0.7899	0.6349	0.6333	0.5970	**0.5253**	**0.5525**	0.5945
CSIQ														
**SRCC**	0.9423	0.9242	0.9310	0.9108	0.9466	0.9133	0.9319	0.9195	0.9582	0.9627	0.9494	**0.9658**	**0.9733**	0.9624
**KRCC**	0.7857	0.7567	0.7690	0.7374	0.7970	0.7393	0.7725	0.7537	0.8165	0.8288	0.7994	**0.8396**	**0.8579**	0.8323
**PCC**	0.9279	0.9120	0.9192	0.8964	0.9500	0.8991	0.9250	0.9277	0.9576	0.9643	0.8968	0.9665	**0.9780**	**0.9704**
**RMSE**	0.0979	0.1077	0.1034	0.1164	0.0820	0.1149	0.0997	0.0980	0.0757	0.0695	0.1162	0.0674	**0.0547**	**0.0634**
LIVE														
**SRCC**	0.9524	0.9634	0.9645	0.9561	0.9669	0.9513	0.9618	0.9636	0.9599	0.9649	0.9627	0.9710	**0.9722**	**0.9749**
**KRCC**	0.8058	0.8337	0.8363	0.8150	0.8421	0.8045	0.8299	0.8282	0.8254	0.8365	0.8281	0.8484	**0.8526**	**0.8600**
**PCC**	0.9482	0.9597	0.9613	0.9512	**0.9675**	0.9489	0.9553	0.9411	0.9586	0.9632	0.8463	0.9580	0.9662	**0.9757**
**RMSE**	8.6816	7.6781	7.5297	8.4327	**6.9073**	8.6188	8.0813	9.2402	7.7765	7.3461	14.5553	7.8351	7.0457	**5.9821**
Overall direct														
**SRCC**	**0.9223**	0.8924	0.9076	0.8780	0.8820	0.8762	0.8963	0.8273	0.9195	0.9139	**0.9306**	0.9171	0.9217	0.9088
**KRCC**	0.7555	0.7285	0.7427	0.7094	0.7218	0.7013	0.7372	0.6707	0.7553	0.7529	**0.7718**	0.7691	**0.7758**	0.7567
**PCC**	0.9131	0.9011	0.9084	0.8840	0.8937	0.8815	0.9065	0.8627	0.9191	0.9199	0.8882	0.9110	**0.9304**	**0.9269**
**RMSE**	0.4283	0.4650	0.4487	0.5000	0.5089	0.5061	0.4517	0.5578	0.4338	0.4376	**0.4054**	0.4459	**0.4091**	0.4266
Overall weighted														
**SRCC**	**0.9103**	0.8598	0.8851	0.8458	0.8412	0.8424	0.8628	0.7657	0.8988	0.8877	**0.9183**	0.8823	0.8895	0.8722
**KRCC**	**0.7370**	0.6898	0.7107	0.6738	0.6711	0.6622	0.6980	0.6061	0.7220	0.7121	**0.7524**	0.7223	0.7295	0.7065
**PCC**	**0.9036**	0.8829	0.8931	0.8653	0.8625	0.8599	0.8875	0.8252	0.9004	0.8981	0.8982	0.8745	**0.9061**	0.9019
**RMSE**	**0.5025**	0.5566	0.5333	0.5932	0.6150	0.6050	0.5456	0.6779	0.5226	0.5313	**0.4703**	0.5706	0.5098	0.5249

The best two IQA models for each criterion are shown in boldface. Overall results for RMSE do not take into account LIVE dataset due to range difference.

The obtained results show that LCSIM3 clearly outperformed other measures, since it yielded the best results on LIVE and CSIQ. It was also the second best measure on TID2008 dataset, after LCSIM2. LSIM1 outperformed other measures on TID2013. Overall results are biased towards techniques that performed well on TID2013, which is the largest benchmark, i.e., LCSIM1, VSI, and IFS. Among results obtained by measures that took part in the LCSIM1 fusion, VSI and MAD are worth noticing. Such good performance of LCSIM family should be confirmed using statistical significance tests. In order to evaluate statistical significance of obtained IQA models, hypothesis tests based on the prediction residuals of each measure after non-linear mapping were conducted using left-tailed F-test [17]. In the test, smaller residual variance denoted the better prediction. Table 3 presents results of these tests, where a symbol “1”, “0” or “-1” denotes that the IQA fusion measure in the row is statistically better with a confidence greater than 95%, indistinguishable, or worse than the IQA measure in the column.

**Table 3:** Statistical significance tests.

VSI	FSIM	FSIMc	GSM	MAD	MSSIM	SR-SIM	VIF	IFS	SFF	LCSIM1	LCSIM2	LCSIM3	LCSIM4	
TID2013														
1	1	1	1	1	1	1	1	1	1	0	1	1	1	**LCSIM1**
-1	-1	-1	-1	-1	-1	-1	1	-1	-1	-1	0	-1	-1	**LCSIM2**
-1	-1	-1	1	1	1	1	1	0	-1	-1	1	0	-1	**LCSIM3**
-1	0	-1	1	1	1	0	1	-1	0	-1	1	1	0	**LCSIM4**
TID2008														
1	1	1	1	1	1	1	1	1	1	0	-1	-1	-1	**LCSIM1**
1	1	1	1	1	1	1	1	1	1	1	0	1	1	**LCSIM2**
1	1	1	1	1	1	1	1	1	1	1	-1	0	1	**LCSIM3**
1	1	1	1	1	1	0	1	1	1	1	-1	-1	0	**LCSIM4**
CSIQ														
1	1	1	1	-1	1	1	1	-1	-1	0	-1	-1	-1	**LCSIM1**
1	1	1	1	1	1	1	1	1	0	1	0	-1	-1	**LCSIM2**
1	1	1	1	1	1	1	1	1	1	1	1	0	1	**LCSIM3**
1	1	1	1	1	1	1	1	1	1	1	1	-1	0	**LCSIM4**
LIVE														
-1	-1	-1	-1	-1	-1	-1	1	-1	-1	0	-1	-1	-1	**LCSIM1**
1	0	0	1	-1	1	-1	1	0	-1	1	0	-1	-1	**LCSIM2**
1	1	1	1	0	1	1	1	1	1	1	1	0	-1	**LCSIM3**
1	1	1	1	1	1	1	1	1	1	1	1	1	0	**LCSIM4**

The fusion measure in the row is significantly better than the IQA measure in the column (’1’), worse (’-1’), or indistinguishable (’0’).

Significance tests confirm good performance of the developed family of multimeasures. LCSIM3 was significantly better than other measures on TID2013, LIVE and CSIQ databases. Its results on TID2013 were also good. However, since it was developed using information carried by scores being a reflectance of the dataset which do not contain many of distortions that are present in CSIQ benchmark, its opinion scores were less correlated in this case than scores of VSI, FCSIM, or IFS. Consequently, LCSIM that was obtained on TID2013 (LCSIM1) performed worse than other measures on LIVE benchmark.

**Fig 2:**
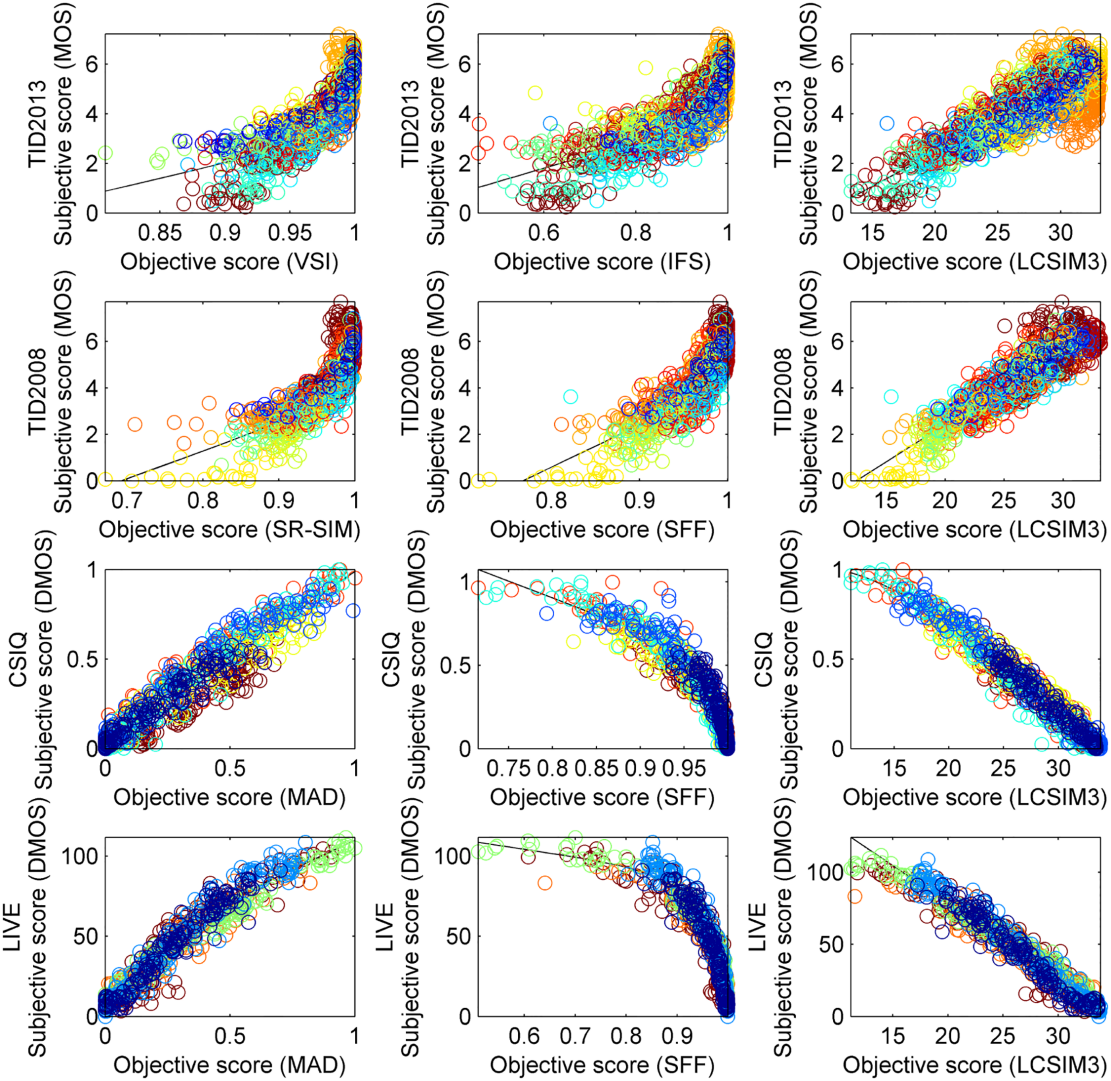
Scatter plots of subjective opinion scores against scores obtained by the two best IQA measures and LCSIM3 on used datasets. Different types of distortions are represented by different colours; the set of colours is coherent within a dataset. Curves fitted with logistic functions are also shown.

**Fig 3:**
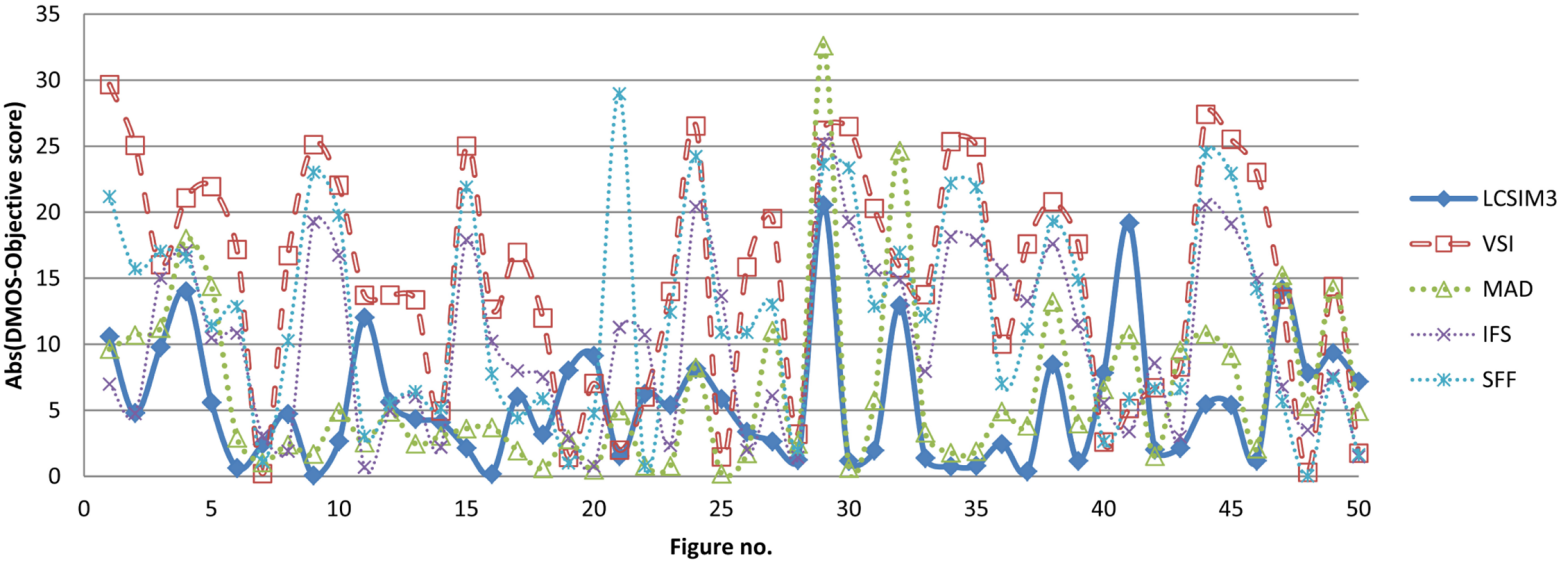
Absolute values of the difference between objective scores and nonlinearly fitted subjective scores for 50 exemplary images from LIVE dataset. For each image, a smaller value denotes objective assessment which is closer to human evaluation.

Fig 2 presents the scatter plots for LCSIM3 and the two best performing IQA models for each benchmark. It can be seen that compared models for databases other than TID2013 yielded less accurate quality predictions for large DMOS values and small MOS values (i.e., in presence of severe distortions) than LCSIM3. Fig 3, in turn, contains absolute values of the difference between subjective scores and objective scores for the five best IQA measures after nonlinear fitting (Eq (3)). Here, the values were obtained for 50 images from the most popular LIVE dataset. The figure shows how scores obtained by IQA measures differ from the expected scores; smaller values are considered better. It can be seen that the introduced fusion measure, LCSIM3, returned scores which are visibly closer to subjective scores obtained in tests with human subjects. This is also confirmed by RMSE values reported for this dataset.

The proposed family of multimeasures aggregates different IQA measures. Therefore, it is worth examining their time- and memory-consumption. The processing time and memory requirements have been determined for all aggregated IQA measures assessing an exemplary image from TID2013 dataset. The results are shown in Table 4. It can be seen that MAD and VIF are the most demanding techniques. Taking into account that processing time requirements for image quality assessment algorithms are less demanding than for video quality assessment techniques, obtained timings on ordinary 2200MHz CPU seem to be acceptable. LCSIMs aggregate several IQA measures; therefore, their running time will be longer in case of sequential execution of used measures or close to the execution time of MAD measure in case of more memory-consuming parallel implementation.

**Table 4:** Time and memory costs of IQA measures used in the optimisation.

IQA measure	Time [s]	Memory [MB]
VSI	0.2207	98.01
FSIM	0.2835	73.62
FSIMc	0.2835	73.62
GSM	0.1796	43.20
IFC	0.8102	356.18
IW-SSIM	0.7160	227.27
MAD	1.1032	725.74
MSSIM	0.1402	33.80
NQM	0.3159	135.55
PSNR	0.0343	1.50
RFSIM	0.0801	3.01
SR-SIM	0.0336	6.95
SSIM	0.0958	17.43
VIF	0.9461	343.89
IFS	0.1213	21.73
SFF	0.0971	17.63

It would be desirable to compare the proposed multimeasures with other related fusion IQA measures. Table 5 contains such comparative evaluation based on SRCC values. SRCC was used as a basis for comparison since many papers do not report other performance indices. Two best results for a given benchmark dataset are written in boldface, some results were not reported in referred works; therefore, they are denoted by “-”. IQA measures which were developed using images from the benchmark in the column are excluded from the comparison. Moreover, overall results were calculated excluding TID2013 since some measures have not been evaluated on it. Furthermore, in order to provide fair comparison, overall results exclude works in which authors obtained a separate IQA measure for each benchmark without providing cross-database evaluation, e.g., [18, 19, 21–23], or [37]. Results for approaches that are not dataset independent are written in italics.

**Table 5:** Comparison of the approach with other fusion IQA measures based on SRCC values.

IQA measure	TID2013	TID2008	CSIQ	LIVE	Overall direct	Overall weighted
**MAD** [17]	0.7807	0.8340	0.9466	0.9669	0.9158	0.8944
**CQM** [18]	-	*0.8720*	-	-	-	-
**Lahouhou et al**. [21]	-	-	-	*0.9500*	-	-
**ADM** [38]	-	0.8617	0.9333	0.9460	0.9137	0.9001
**BMMF** [39]	0.8340	*0.9471*	-	-	-	-
**BME** [22]	-	*0.8882*	*0.9573*	*0.9711*	-	-
**RMSSIM** [23]	-	*0.8569*	*0.9453*	*0.9633*	-	
**IGM** [40]	-	0.8902	0.9401	0.9580	0.9294	0.9190
**EHIS** [19]	-	*0.9098*	*0.9498*	*0.9622*	-	-
**MMF** [37]	-	*0.9487*	*0.9755*	*0.9732*	-	-
**GLD-PFT** [24]	-	0.8849	0.9549	0.9631	0.9343	0.9186
**Barri et al.** [41]	-	0.8100	**0.9630**	*0.9570*	0.9100	0.8843
**DOG-SSIM** [42]	**0.8942**	*0.9259*	0.9204	0.9423	0.9295	0.9282
**ESIM** [20]	**0.8804**	*0.9026*	0.9620	0.9420	0.9420	0.9300
**LCSIM1**	*0.9044*	**0.9057**	0.9494	0.9627	0.9393	0.9280
**LCSIM2**	0.8139	*0.9178*	**0.9658**	**0.9710**	**0.9515**	**0.9408**
**LCSIM3**	0.8307	**0.9107**	*0.9733*	**0.9722**	**0.9521**	**0.9395**
**LCSIM4**	0.8086	0.8892	0.9624	*0.9749*	0.9422	0.9251

The result for the measure that was trained using images from the dataset indicated in the column is italicised in order to show the lack of the dataset independence. Overall results were calculated on the basis of three most popular datasets (i.e., TID2008, CSIQ and LIVE) taking into account IQA measures that provided independent results for at least two datasets. The two best measures for each dataset are shown in boldface.

Evaluation results show that LCSIM3 and LCSIM2 outperformed other approaches which use fusion of IQA measures. Among other measures, DOG-SSIM and ESIM provided good results on TID2013 benchmark, and the approach developed by Barri et al. turned out to be the second best technique on CSIQ dataset. Outstanding performances of LCSIM3 and LCSIM2 are also confirmed by overall results. Here, they are followed by ESIM, LCSIM4, LCSIM1, and DOG-SIM. Most of these models were trained on TID2008, except LCSIM3 that was trained on images from CSIQ. This happened since all three most popular datasets share the same types of distortions.

### Influence of parameters and IQA measures on fusion

The already presented results confirm good performance of obtained IQA fusion measures in comparison with state-of-the-art fusion and single IQA measures. However, it would be desirable to answer why some measures took part in the fusion more often than others. A contribution of aggregated models also requires some attention, since the linear combination can produce unintuitive negative weights.

At first, in order to show the contribution of a given measure, SRCC values between objective and subjective scores were obtained for each distortion type. This may explain why some measures were involved in a fusion, and also show how well perform developed LCSIMs in comparison with IQA measures that were used in optimisation, from distortion type point of view. Table 6 contains SRCC values of the best ten IQA models and LCSIMs obtained on benchmark datasets. The two best IQA measures for each distortion type are written in boldface.

**Table 6:** SROCC values of IQA measures for each distortion type.

Dist. Type	VSI	FSIM	FSIMc	GSM	MAD	MSSIM	SR-SIM	VIF	IFS	SFF	LCSIM1	LCSIM2	LCSIM3	LCSIM4
TID2013														
**AWGN**	**0.9460**	0.8973	0.9101	0.9064	0.8843	0.8646	0.9212	0.8994	0.9382	0.9066	**0.9389**	0.9248	0.9240	0.9141
**AWGNc**	**0.8705**	0.8208	0.8537	0.8175	0.8019	0.7730	0.8496	0.8299	0.8537	0.8166	**0.8584**	0.8482	0.8481	0.8464
**SCN**	**0.9367**	0.8750	0.8900	0.9158	0.8911	0.8544	0.9150	0.8835	**0.9340**	0.8982	0.9335	0.9253	0.9223	0.9090
**MN**	0.7697	0.7944	0.8094	0.7293	0.7380	0.8073	0.7645	**0.8450**	0.7960	**0.8185**	0.7732	0.8175	0.8021	0.7901
**HFN**	**0.9200**	0.8984	0.9040	0.8869	0.8876	0.8604	0.9102	0.8972	**0.9140**	0.8977	0.9085	0.9081	0.9098	0.9002
**IN**	**0.8741**	0.8072	0.8251	0.7965	0.2769	0.7629	0.8249	**0.8537**	0.8389	0.7871	0.7989	0.7905	0.7218	0.5710
**QN**	0.8748	0.8719	**0.8807**	**0.8841**	0.8514	0.8706	0.8447	0.7854	0.8335	0.8607	0.8787	0.8523	0.8721	0.8574
**GB**	0.9612	0.9551	0.9551	**0.9689**	0.9319	0.9673	0.9612	0.9650	0.9658	**0.9675**	0.9588	0.9623	0.9642	0.9503
**DEN**	**0.9484**	0.9302	0.9330	**0.9432**	0.9252	0.9268	0.9371	0.8911	0.9183	0.9091	0.9423	0.9194	0.9307	0.9144
**JPEG**	**0.9541**	0.9324	0.9339	0.9284	0.9217	0.9265	**0.9398**	0.9192	0.9290	0.9273	0.9346	0.9234	0.9256	0.9274
**JP2K**	**0.9706**	0.9577	0.9589	0.9602	0.9511	0.9504	**0.9655**	0.9516	0.9611	0.9571	0.9627	0.9605	0.9614	0.9574
**JGTE**	**0.9216**	0.8464	0.8610	0.8512	0.8283	0.8475	0.8527	0.8409	**0.8925**	0.8831	0.8917	0.8626	0.8607	0.8540
**J2TE**	**0.9228**	0.8913	0.8919	0.9182	0.8788	0.8889	0.9047	0.8761	0.9010	0.8708	0.9184	0.9194	**0.9204**	0.9008
**NEN**	0.8060	0.7917	0.7937	0.8130	**0.8315**	0.7968	0.7617	0.7720	0.7839	0.7668	0.8100	0.8129	0.8166	**0.8286**
**BWD**	0.1713	0.5489	**0.5532**	**0.6418**	0.2812	0.4801	0.4571	0.5306	0.1004	0.1786	0.3522	0.3295	0.1854	0.4517
**IS**	0.7700	0.7531	0.7487	**0.7875**	0.6450	**0.7906**	0.6402	0.6276	0.6575	0.6654	0.6778	0.4537	0.6150	0.5572
**CC**	0.4754	0.4686	0.4679	0.4857	0.1972	0.4634	0.4644	**0.8386**	0.4469	0.4691	0.4445	**0.6286**	0.4253	0.5324
**CCS**	0.8100	0.2748	**0.8359**	0.3578	0.0575	0.4099	0.1875	0.3099	0.8257	0.8269	**0.8324**	0.8094	0.8171	0.7155
**MGN**	**0.9117**	0.8469	0.8569	0.8348	0.8409	0.7786	0.8719	0.8468	0.8790	0.8434	**0.8952**	0.8830	0.8812	0.8746
**CN**	**0.9243**	0.9121	0.9135	0.9124	0.9064	0.8528	0.9199	0.8946	0.9037	0.9007	**0.9180**	0.9116	0.9127	0.9074
**LCNI**	0.9564	0.9466	0.9485	0.9563	0.9443	0.9068	**0.9591**	0.9204	0.9433	0.9262	**0.9600**	0.9503	0.9498	0.9436
**ICQD**	0.8839	0.8760	0.8815	0.8973	0.8745	0.8555	0.8727	0.8414	**0.9007**	0.8795	**0.9019**	0.8840	0.8983	0.8825
**CHA**	**0.8906**	0.8715	**0.8925**	0.8823	0.8310	0.8784	0.8746	0.8848	0.8862	0.8789	0.8805	0.8765	0.8736	0.8611
**SSR**	0.9628	0.9565	0.9576	**0.9668**	0.9567	0.9483	0.9613	0.9353	0.9556	0.9522	**0.9656**	0.9585	0.9619	0.9545
TID2008														
**AWGN**	**0.9229**	0.8566	0.8758	0.8606	0.8386	0.8086	0.8990	0.8797	**0.9172**	0.8731	0.9087	0.9147	0.8998	0.8803
**AWGNc**	**0.9118**	0.8527	0.8931	0.8091	0.8255	0.8054	0.8953	0.8757	0.8958	0.8626	0.8928	**0.8993**	0.8897	0.8852
**SCN**	0.9296	0.8483	0.8711	0.8941	0.8678	0.8209	0.9083	0.8698	**0.9307**	0.8939	0.9224	**0.9330**	0.9187	0.8967
**MN**	0.7734	0.8021	0.8264	0.7452	0.7336	0.8107	0.7870	**0.8683**	0.8021	0.8365	0.7288	**0.8530**	0.8181	0.7822
**HFN**	**0.9253**	0.9093	0.9156	0.8945	0.8864	0.8694	0.9197	0.9075	**0.9215**	0.9119	0.9108	0.9190	0.9205	0.9023
**IN**	**0.8298**	0.7452	0.7719	0.7235	0.0650	0.6907	0.7665	**0.8327**	0.8143	0.7484	0.7325	0.7524	0.6178	0.3996
**QN**	**0.8731**	0.8564	0.8726	**0.8800**	0.8160	0.8589	0.8364	0.7970	0.7973	0.8448	0.8608	0.8541	0.8536	0.8297
**GB**	0.9529	0.9472	0.9472	0.9600	0.9196	0.9563	0.9549	0.9540	**0.9602**	**0.9624**	0.9519	0.9574	0.9568	0.9405
**DEN**	**0.9693**	0.9603	0.9618	**0.9725**	0.9433	0.9582	0.9668	0.9161	0.9491	0.9383	0.9633	0.9594	0.9569	0.9424
**JPEG**	**0.9616**	0.9279	0.9294	0.9393	0.9275	0.9322	0.9394	0.9168	0.9279	0.9323	0.9340	**0.9444**	0.9344	0.9257
**JP2K**	**0.9848**	0.9773	0.9780	0.9758	0.9707	0.9700	0.9807	0.9709	0.9778	0.9772	0.9821	**0.9825**	0.9796	0.9768
**JGTE**	**0.9160**	0.8708	0.8756	0.8790	0.8661	0.8681	0.8881	0.8585	0.8735	0.8567	**0.9123**	0.8984	0.8987	0.8938
**J2TE**	0.8942	0.8544	0.8555	0.8936	0.8394	0.8606	0.8903	0.8501	0.8799	0.8386	0.8925	**0.9137**	**0.9005**	0.8816
**NEN**	0.7699	0.7491	0.7514	0.7386	**0.8287**	0.7377	0.7670	0.7619	0.7035	0.6970	0.7770	0.8031	0.7985	**0.8238**
**BWD**	0.6295	**0.8492**	0.8464	**0.8862**	0.7970	0.7546	0.7787	0.8324	0.0871	0.5369	0.6329	0.7376	0.6665	0.8259
**IS**	0.6714	0.6720	0.6554	**0.7190**	0.5163	**0.7336**	0.5728	0.5096	0.5215	0.5225	0.3808	0.5587	0.4928	0.4602
**CC**	0.6557	0.6481	0.6510	0.6691	0.2723	0.6381	0.6483	**0.8188**	0.6273	0.6461	0.6096	**0.7258**	0.5764	0.6323
CSIQ														
**AWGN**	0.9636	0.9262	0.9359	0.9440	0.9541	0.9471	0.9628	0.9575	0.9593	0.9467	0.9618	**0.9717**	**0.9718**	0.9625
**JPEG**	0.9618	0.9654	0.9664	0.9632	0.9615	0.9634	0.9671	0.9705	0.9660	0.9641	0.9206	**0.9711**	**0.9708**	0.9570
**JP2K**	0.9694	0.9685	0.9704	0.9648	0.9752	0.9683	0.9773	0.9672	0.9712	0.9763	0.9759	**0.9781**	**0.9791**	0.9731
**AGPN**	0.9638	0.9234	0.9370	0.9387	0.9570	0.9331	0.9520	0.9511	0.9526	0.9550	**0.9668**	0.9650	**0.9657**	0.9650
**GB**	0.9679	0.9729	0.9729	0.9589	0.9682	0.9711	0.9767	0.9745	0.9621	0.9751	0.9640	**0.9790**	**0.9774**	0.9735
**GCD**	0.9504	0.9420	0.9438	0.9354	0.9207	0.9526	0.9528	0.9345	0.9485	**0.9536**	0.9510	**0.9590**	0.9443	0.9449
LIVE														
**JP2K**	0.9604	0.9717	**0.9724**	0.9700	0.9676	0.9627	0.9701	0.9696	0.9694	0.9672	0.9695	0.9690	0.9682	**0.9721**
**JPEG**	0.9761	0.9834	**0.9840**	0.9778	0.9764	0.9815	0.9823	**0.9846**	0.9778	0.9786	0.9761	0.9823	0.9792	0.9818
**AWGN**	0.9835	0.9652	0.9716	0.9774	0.9844	0.9733	0.9810	0.9858	**0.9883**	0.9859	0.9855	0.9878	**0.9884**	0.9873
**GB**	0.9527	0.9708	0.9708	0.9518	0.9465	0.9542	0.9660	**0.9728**	0.9665	**0.9752**	0.9664	0.9641	0.9640	0.9589
**FF**	0.9430	0.9499	0.9519	0.9402	0.9569	0.9471	0.9465	**0.9650**	0.9404	0.9529	0.9499	0.9604	0.9616	**0.9694**

Results for distortion types reveal that VSI, FSIMc, GSM, VIF, IFS, and SFF are among best single IQA models. They also were often a part of fusion models, what can be seen in Eqs (9)–(12). Here, LCSIM family was better or close to best IQA models and showed outstanding performance on CSIQ dataset. In order to provide further investigation why some measures were fused together, SRCC values between IQA models on CSIQ dataset were obtained. They are shown in Table 7. This time correlation sign was preserved, since it may suggest why some measures have negative weights in fusions. Negative correlations can also be seen on Fig 2. Similar pairwise relations between IQA models were noticed on other datasets. It can be seen that some measures are less correlated with each other while preserving good correlation with subjective scores. VIF is the less correlated measure with MSSIM and MAD, all these measures perform well on CSIQ dataset. SRCC values for these measures are written in boldface in the Table 7. IQA measures in pairs MAD—VIF and MSSIM—VIF are complementary and thus likely to be fused together.

**Table 7:** SRCC between objective scores of IQA measures on CSIQ.

	VSI	FSIM	FSIMc	GSM	MAD	MSSIM	SR-SIM	VIF	IFS	SFF
**VSI**		0.9722	0.9808	0.9719	-0.9563	0.9743	0.9706	0.8813	0.9684	0.9603
**FSIM**	0.9722		0.9985	0.9884	-0.9613	0.9852	0.9865	0.8879	0.9462	0.9436
**FSIMc**	0.9808	0.9985		0.9880	-0.9662	0.9877	0.9850	0.8868	0.9552	0.9515
**GSM**	0.9719	0.9884	0.9880		-0.9548	0.9857	0.9831	0.8536	0.9336	0.9271
**MAD**	-0.9563	-0.9613	-0.9662	-0.9548		**-0.9605**	-0.9552	**-0.8689**	-0.9422	-0.9451
**MSSIM**	0.9743	0.9852	0.9877	0.9857	-0.9605		0.9719	0.8538	0.9358	0.9292
**SR-SIM**	0.9706	0.9865	0.9850	0.9831	-0.9552	0.9719		0.9010	0.9414	0.9422
**SSIM**	0.9382	0.9602	0.9603	0.9655	-0.9318	0.9675	0.9428	0.8278	0.8986	0.8863
**VIF**	0.8813	0.8879	0.8868	0.8536	-0.8689	**0.8538**	0.9010		0.9233	0.9276
**IFS**	0.9684	0.9462	0.9552	0.9336	-0.9422	0.9358	0.9414	0.9233		0.9916
**SFF**	0.9603	0.9436	0.9515	0.9271	-0.9451	0.9292	0.9422	0.9276	0.9916	
**Mean**	0.7662	0.7707	0.7727	0.7642	**-0.9442**	**0.7631**	0.7669	**0.7074**	0.7552	0.7514
**SRCC on CSIQ**	-0.9423	-0.9242	-0.9310	-0.9108	**0.9466**	**-0.9133**	-0.9319	**-0.9195**	-0.9582	-0.9627

These findings were confirmed in an experiment in which a predefined number of IQA measures, *k* ∈ *N*, could take part in the fusion. Such reduced fusion models are helpful to determine the contribution of each fused measure. In the experiment, *k* varied from 2 to 5. In order to estimate the influence of the IQA measure on the results obtained by the fusion model, the percentage decrease of RMSE without the measure was calculated. Table 8 contains such reduced LCSIMs for CSIQ dataset, their RMSE values, and contributions. The table also contains LCSIM3, since it was developed on images from CSIQ.

**Table 8:** Results of the experiment with predefined number of aggregated IQA models on CSIQ dataset.

No. of IQA models, *k*	Equation	RMSE	Contribution [%]
**2**	-6.0533MAD + 3.8434VIF	0.0619	**39.13**, **24.51**
**3**	-1.8294MAD + 1.1814VIF + 8.7912SFF	0.0592	**27.63**, **94.36**, 5.58
**4**	-11.8203GSM—3.3564MAD + 2.0515VIF + 3.5018IFS	0.0587	2.65, **32.06**, **19.81**, 8.71
**5**	-3.6729FSIMc—4.5873MAD + 2.5546VIF + 4.8361IFS + 12.0375SFF	0.0574	3.20, **26.22**, **24.17**, 5.28, 2.55
-	LCSIM3, Eq (11)	0.0547	0.18, 2.32, 1.80, 0.01, **30.85**, **79.75**, 4.04, 6.17, **13.59**, 3.36, 1.08

Contribution is calculated as percentage decrease of RMSE without a given IQA measure. Contributions of aggregated measures are separated with comma; values for MAD, VIF, and MSSIM are written in boldface.

Results shown in Table 8 confirm that the IQA measures that achieve good performance on CSIQ dataset and are less correlated with each other, are likely to be aggregated. In obtained fusion measures, weights do not reflect well the contribution of selected IQA measures, what can be seen in case of three (*k* = 3) fused models, where VIF and MAD with lower weights contributed more than SFF. The sign of the weight depends on correlation of the measure with objective scores (MOS or DMOS) but it can also be used as compensation, making the resulting vector of objective opinion scores closer to the vector of subjective scores, since the optimisation utilise RMSE between them for finding better aggregated models.

It is worth noticing that RMSE results obtained by all measures developed in experiments with the predefined number of IQA measures are better than results of state-of-the-art approaches on this dataset (see Table 8).

MAD, VIF, and MSSIM contributed the most to LCSIM measures obtained on CSIQ dataset. This can also be observed for the remaining LCSIM measures, where the best contributing three IQA single models are as follows: MAD (19.76%), IFS (16.90%), and PSNR (16.71%) to LCSIM1, VIF (15.87%), MAD (8.31%), and SSIM (4.49%) to LCSIM2, VIF (38.54%), MAD (33.87%), and GSM (4.22%) to LCSIM4.

The ***β*** used in calculation of RMSE (and PCC) also influenced the results. In order to show its influence, each *β* component, ***β*** = [*β*_1_, *β*_2_, …, *β*_5_], determined in optimisation for a given LCSIM was changed in the range 0.1 to 20 with the step 0.1, while other components remained unchanged. Table 9 presents minimum, maximum, mean and standard deviation of RMSE values for each component calculated on benchmark datasets. It can be seen that *β*_4_ has the largest influence on LCSIM1, *β*_2_ on LCSIM2, *β*_3_ on LCSIM3, and all components are similarly important to LCSIM4.

**Table 9:** Influence of the β on obtained IQA fusion measures.

*β*	Min	Max	Mean	SD
LCSIM1 on TID 2013				
*β*_1_	0.5030	0.6849	0.5474	0.0451
*β*_2_	0.5030	0.6768	0.5884	0.0087
*β*_3_	0.5030	0.5881	0.5856	0.0145
*β*_4_	0.5030	1.3554	0.5941	0.0810
*β*_5_	0.5030	0.5998	0.5570	0.0412
LCSIM2 on TID 2008				
*β*_1_	0.5253	0.5253	0.5253	0.0000
*β*_2_	0.5253	10.1183	0.5815	0.6780
*β*_3_	0.5253	0.5253	0.5253	0.0000
*β*_4_	0.5253	0.5253	0.5253	0.0000
*β*_5_	0.5253	0.5253	0.5253	0.0000
LCSIM3 on CSIQ				
*β*_1_	0.0547	0.0571	0.0554	0.0008
*β*_2_	0.0547	0.0571	0.0556	0.0008
*β*_3_	0.0547	0.2053	0.0625	0.0287
*β*_4_	0.0547	0.0571	0.0557	0.0009
*β*_5_	0.0547	0.0571	0.0552	0.0005
LCSIM4 on LIVE				
*β*_1_	5.9820	6.4776	5.9976	0.0387
*β*_2_	5.9820	6.0315	6.0024	0.0187
*β*_3_	5.9820	6.9150	6.0253	0.0836
*β*_4_	5.9820	6.0320	6.0136	0.0185
*β*_5_	5.9820	6.0016	5.9837	0.0054

## Conclusions

In this paper, a multimeasure resulted from a fusion of full-reference IQA measures is presented. The fusion was formulated as an optimisation problem that was solved using the genetic algorithm, which was also responsible for selection of appropriate IQA measures. Evaluation of the proposed approach on widely used four largest image benchmarks reveals that LCSIM family of measures performs better than compared state-of-the-art IQA models, in terms of prediction quality reflected by SRCC, KRCC, PCC, and RMSE. The contribution of aggregated IQA measures was also investigated in the paper.

Further extension of the approach could involve using other IQA measures for fusion; therefore, Matlab source code that would allow running the optimisation with any newly developed measure with known objective scores for used image benchmarks and evaluate the results, is available to download at http://marosz.kia.prz.edu.pl/LCSIM.html. Another direction of future research would be to develop a fusion measure oriented on a given type of distortion or a measure which aggregates full-reference IQA measures with small memory footprint and short computation time.
